# Variation in the vitamin D receptor gene, plasma 25-hydroxyvitamin D, and risk of premenstrual symptoms

**DOI:** 10.1186/s12263-021-00696-2

**Published:** 2021-09-22

**Authors:** Alicia C. Jarosz, Daniel Noori, Tara Zeitoun, Bibiana Garcia-Bailo, Ahmed El-Sohemy

**Affiliations:** Department of Nutritional Sciences, Temerty Faculty of Medicine, University of Toronto, Ontario M5S 1A8 Toronto, Canada

**Keywords:** Vitamin D, VDR, Vitamin D receptor, Premenstrual symptoms, Nutrigenomics, Nutrigenetics

## Abstract

**Background:**

Vitamin D status has been associated with the presence and severity of several premenstrual symptoms (PMSx) in some, but not all studies. Inconsistencies among findings may be explained by unaccounted genetic variation in the vitamin D receptor (*VDR*).

**Objective:**

To determine whether associations between vitamin D status and individual PMSx are influenced by *VDR* genotype.

**Methods:**

Seven hundred sixteen women aged 20-29 years old from the Toronto Nutrigenomics and Health study provided plasma samples and completed a questionnaire on the presence and severity of 15 common PMSx. Plasma 25-hydroxyvitamin D (25(OH)D) concentration was measured and participants were categorized into sufficient (≥ 50 nmol/L) and insufficient (< 50 nmol/L) vitamin D status groups. DNA was obtained from blood samples to genotype for a common *VDR* single nucleotide variant, rs796858. Using logistic regression, odds of experiencing PMSx were compared between vitamin D-sufficient and insufficient women, stratified by genotype.

**Results:**

Among CC homozygotes, insufficient vitamin D status was associated with higher odds of experiencing premenstrual fatigue (OR, 2.53; 95% CI, 1.40, 4.56) and nausea (OR, 2.44; 95% CI, 1.00, 5.95). Among TT homozygotes, insufficient vitamin D status was associated with lower odds of experiencing fatigue (OR, 0.44; 95% CI, 0.20, 0.97) and increased appetite (OR, 0.48; 95% CI, 0.22, 1.04). Insufficient vitamin D status was associated with higher odds of increased appetite in women with the CT genotype (OR, 1.78; 95% CI, 1.03, 3.07). *VDR* genotype modified the association between vitamin D status and the following PMSx: increased appetite (interaction *p* = 0.027), fatigue (interaction *p* = 0.016), and nausea (interaction *p* = 0.039).

**Conclusion:**

We found evidence that *VDR* genotype may modify the association between 25(OH)D and some PMSx. Insufficient 25(OH)D was associated with a higher risk of premenstrual fatigue in those with the CC genotype, but lower risk in those with the TT genotype.

**Supplementary Information:**

The online version contains supplementary material available at 10.1186/s12263-021-00696-2.

## Introduction

Premenstrual symptoms (PMSx) occur during the late luteal phase of the menstrual cycle and resolve once menstruation begins [1]. It is estimated that 85-98% of women experience these symptoms [2], but the type of symptoms and the severity differ between women. Moderate-severe symptoms are debilitating and can significantly impact the quality of life and productivity of those who experience them [3–7]. There are currently two distinctly recognized premenstrual disorders (PMDs): premenstrual syndrome (PMS) and premenstrual dysphoric disorder (PMDD). These PMDs are estimated to affect 20-32% and 3-8% of women, respectively. The definition for their diagnosis is based on symptom number and type (somatic or affective) [2, 8]. As such, a PMD diagnosis often conveys little about the underlying etiology of the many different PMSx that a woman can experience. Studying PMSx individually can offer better insights into pathophysiological mechanisms and strategies for their prevention and management.

The etiology of PMSx is inexact; however, lifestyle factors, genetics, and nutrition are all key features in the variation of PMSx [9–11]. The role of vitamin D in PMSx is not yet fully understood as some studies have indicated inverse associations, while other studies report no associations between vitamin D intake or plasma levels and the risk of PMSx severity [11, 12]. Evidence from a recent meta-analysis indicates that vitamin D supplementation is effective in treating PMS, but it is not clear which specific symptoms are alleviated [13]. Plasma 25(OH)D concentrations have also been shown to be inversely associated with the presence or severity of some PMSx [14, 15]. Though, the exact mechanism behind these findings remains equivocal, as there is no evidence to suggest that vitamin D metabolites differ during different phases of the menstrual cycle [16]. Plasma 25(OH)D is the most abundant circulating vitamin D metabolite; to exert biological effects, 25(OH)D must be converted into the active 1,25-dihydroxyvitamin D (1,25(OH)D), which binds to the vitamin D receptor (VDR) [17–19]. 25(OH)D is a more accurate measure of physiological vitamin D availability than dietary intake, as it encompasses cutaneous vitamin D production in addition to intake.

The biological effects of vitamin D are now believed to transcend the vitamin’s classic role in bone health because the VDR binds to response elements in the regulatory region of at least 900 genes [20]. Indeed, many cell types express the VDR, suggesting a plausible involvement of vitamin D in regulating the physiological processes involved in PMSx [21]. More specifically, previous findings suggest that variants in the *VDR* gene, such as the rs7968585 single nucleotide polymorphism (SNP) modify the association between low vitamin D concentration and risk of major health outcomes [22, 23]. Additionally, polymorphisms in the *VDR* gene may alter these effects on target genes that are induced by the binding of 1,25(OH)D, influencing the body’s response to vitamin D and potentially modifying the association between vitamin D status and PMSx. Considering that there is evidence for genetic heritability in PMS, and that the relationship between vitamin D and PMSx remains equivocal, the objective of the present study was to determine whether associations between vitamin D status and individual PMSx may be influenced by genetic variation in *VDR*.

## Methods

### Study population

Subjects were participants of the Toronto Nutrigenomics and Health (TNH) study, which is a cross-sectional analysis of a multiethnic population of young adults. The study recruited 1636 healthy men and women aged 20-29 years and living in Toronto, Canada, between the years 2004 and 2010. Women who were pregnant or breastfeeding were not eligible to participate. Participants who met inclusion criteria provided written informed consent, and the study protocol was approved by the Ethics Review Board of the University of Toronto.

For the present study, male participants (*n* = 520), participants missing General Health and Lifestyle Questionnaire (GHLQ) or food frequency questionnaire (FFQ) data (*n* = 26), and participants missing biomarker or genetic data (*n* = 7) were excluded. Participants using hormonal contraceptives (HCs) (*n* = 275), anxiolytics or anti-depressants (*n* = 45), and current or past smokers (*n* = 50) were also excluded from the present analyses due to the confounding effects of these variables on vitamin D status or premenstrual symptoms [45, 46]. The remaining 716 participants were categorized into four major ethnic groups based on their self-reported ethnocultural status: Caucasian (*n* = 254), East Asian (*n* = 330), South Asian (*n* = 82), and other (*n* = 50). Caucasians included those self-reported as European, Middle Eastern, or Hispanic. East Asians consisted of Chinese, Korean, Japanese, Filipino, Vietnamese, Thai, and Cambodian. South Asians included Bangladeshi, Sri Lankan, Indian, and Pakistani. The other category included Aboriginal Canadians, Afro-Caribbeans, and those who self-reported belonging to ≥ 2 ethnic groups.

### General health and lifestyle questionnaire

The GHLQ included questions regarding age, gender, ethnocultural group, current medical conditions, medication use, and hormonal contraceptive use, as well as dietary supplements, special diets, and physical activity. A PMSx questionnaire was included in the GHLQ, which assessed the presence and severity of 15 commonly reported PMSx within 5 days prior to the onset of menses and up to 4 days thereafter, as described previously [24, 25]. Severity categories included none, mild, moderate, and severe. In the present study, severities were grouped into present (mild, moderate, severe) and absent (none). Participants were presented with the option of listing other symptoms not included in the questionnaire; however, very few additional symptoms were reported this way and they were not considered in the analyses in the present study.

### Anthropometric and plasma 25(OH)D measurements

Height and weight were measured by trained study personnel and used to calculate body mass index (BMI) (weight (kg)/height (m)^2^). The subjects self-reported their physical activity in the GHLQ by providing an estimate of the amount of time they spent sleeping and partaking in light, moderate, and vigorous activity. These values were subsequently converted into metabolic equivalent (MET) hours per week.

Following a 12-h overnight fast, blood samples were collected at LifeLabs Medical Laboratory Services (Toronto, Ontario, Canada). Participants experiencing a temporary inflammatory condition (a recent piercing or tattoo, acupuncture, immunization or vaccination, flu, fever, medical or dental procedure, infection) provided blood samples following a 2-week recovery period. Plasma 25(OH)D was measured using high-performance liquid chromatography-tandem mass spectrometry at the University Health Network Specialty Lab at Toronto General Hospital, as previously described [26]. Reported plasma 25(OH)D concentrations are the sum of measured 25(OH)D_3_ and 25(OH)D_2_.

The season of blood draw was classified as follows based on month of blood draw: spring (March, April, May), summer (June, July, August), fall (September, October, November), and winter (December, January, February). Vitamin D status categories were created based on recommendations from the Canadian Osteoporosis Society, the Endocrine Society, and the Institute of Medicine [27–29]. Insufficient and sufficient vitamin D status categories were defined as 25(OH)D < 50 nmol/L and 25(OH)D ≥ 50 nmol/L, respectively.

### Dietary assessment

Total calcium intake, which can affect PMSx [30], was measured using a 196-item Toronto-Modified Harvard FFQ that assessed subjects’ dietary intake of foods, beverages, and supplements. Subjects estimated their consumption of a preassigned portion of each item over the past month by choosing from several frequency options. Responses were subsequently converted into estimate daily averages of total calcium intake from foods and supplements.

### Genotyping

Blood samples were processed at the Clinical Genomics Centre at Princess Margaret Hospital, University Health Network (Toronto, Ontario, Canada) and were genotyped for rs7968585 in the *VDR* gene using the iPLEX Gold assay with MS-based detection (Sequenom MassARRAY platform; Sequenom, Inc.) [31]. This specific polymorphism in the VDR gene was selected because previous studies have shown that it modifies the association between 25(OH)D and major health outcome risk [22, 23].

### Statistical analysis

All analyses were conducted using the SAS Statistical Analysis Software (version 9.4; SAS Institute Inc., Cary, NC, USA). Sample size was determined using a power of 0.8, a small effect size of 0.2, and a significance of *p* < 0.05 using R (version 4.0.3) and RStudio (version 1.3.1). Based on this equation, and the observed sample size in previous studies assessing 25(OH)D, an adequate sample size was estimated to be ~190 participants [14, 32]. *P* values were two-sided. Distributions of continuous variables were assessed for normality prior to analyses and adjusted as necessary. Subject characteristics were compared between participants with sufficient and insufficient vitamin D status using ANOVA for continuous variables and chi-square tests for categorical variables. Logistic regressions were performed to determine if *VDR* genotype modified the association between vitamin D status and the presence of PMSx. Odds ratios (OR) and corresponding 95% confidence intervals (CI) were calculated to compare the odds of experiencing PMSx for participants with insufficient versus sufficient vitamin D status, using “no symptoms” as the reference category, stratified by *VDR* genotype. The *p* values for the interaction term between *VDR* and vitamin D status were also calculated for each symptom. Univariable and multivariable analyses were carried out. Covariates included age, ethnicity, BMI, physical activity, season of blood draw, calcium intake, and use of analgesics. Benjamini-Yekutieli adjustments for multiple comparisons were applied (15 tests, *α* = 0.05: *p* < 0.015) to the interaction term *p* values.

## Results

Table 1 displays the subject characteristics stratified by vitamin D status. The average age was 22.4 years, and women with an insufficient vitamin D status were younger than vitamin D-sufficient women (*p* < 0.01). Likewise, average reported physical activity also differed by vitamin D status (*p* < 0.0001), where women with insufficient status reported 7.1 ± 3.1 MET-hours/week of physical activity compared to 8.1 ± 3.2 MET-hours/week in the sufficient status group. The average BMI was 22.3 kg/m^2^; however, this did not significantly differ by vitamin D status (*p* = 0.62). Total vitamin D intake differed by plasma vitamin D status as well (*p* < 0.001), where women with insufficient status reported 279 ± 218 mg/day compared to 403 ± 278 mg/day. In the present study, East Asians (47%) were the most common ethnocultural group, followed by Caucasians (35%), South Asians (11%), and other (7%). Additionally, the distribution of the ethnic groups differed significantly by vitamin D status, with Caucasians having the greatest proportion of individuals classified as vitamin D-sufficient (*p* < 0.0001). The distribution of ethnic groups also differed significantly by *VDR* genotype (*p* < 0.001) (Supplementary Table 1). No significant differences were observed in the distribution of analgesic use or *VDR* genotypes between vitamin D status groups. There were also no significant differences observed between total vitamin D intake and *VDR* genotypes (data not shown).

**Table 1:** Participant characteristics stratified by vitamin D status categories^a^

	Inadequate (< 50 nmol/L)	Adequate (≥ 50 nmol/L)	***p*** value^**b**^
*n* (%)	396 (55)	320 (45)	
Age, (years)	22.2 ± 2.3	22.7 ± 2.6	0.007
Ethnicity, *n* (%)			< 0.0001
Caucasian	87 (34)	167 (66)	
East Asian	221 (67)	109 (33)	
South Asian	65 (79)	17 (21)	
Other	23 (46)	27 (54)	
BMI, (kg/m^2^)^c^	22.3 ± 3.8	22.4 ± 3.1	0.62
Calcium intake, (mg/day)	919 ± 496	1092 ± 472	< 0.0001
Vitamin D intake (mg/day)	279 ± 218	403 ± 278	< 0.001
Physical activity, (MET-h/week)^d^	7.1 ± 3.1	8.1 ± 3.2	< 0.0001
Analgesic use, *n* (%)	95 (56)	74 (43)	0.79
*VDR*^e^ genotype, *n* (%)			0.31
CC	152 (59)	106 (41)	
CT	159 (52)	144 (48)	
TT	85 (55)	70 (45)	

^a^Values are unadjusted means ± standard deviations for continuous variables, unless otherwise indicated

^b^Differences between groups were compared using chi-square tests for categorical variables and ANOVA for continuous variables

^c^*BMI* body mass index

^d^MET metabolic equivalent

^e^*VDR* vitamin D receptor

The associations between vitamin D status and individual PMSx stratified by *VDR* rs7968585 genotype are shown in Table 2. Following adjustment for covariates, *VDR* genotype modified the association between vitamin D status and premenstrual increased appetite (interaction *p* = 0.027), fatigue (interaction *p* = 0.016), and nausea (interaction *p* = 0.039) as shown in Table 3. These interactions should be interpreted with caution since none met the Benjamini-Yekutieli threshold for multiple testing (*p* = 0.015). In analyses stratified by genotype, insufficient vitamin D status was associated with a lower risk fatigue (OR, 0.44; 95% CI, 0.20, 0.97), compared to vitamin D sufficiency in women with the TT genotype. Insufficient vitamin D status was also associated with a nearly significant lower risk of increased appetite, compared to vitamin D sufficiency (OR, 0.48; 95% CI, 0.22, 1.04) in women with the TT genotype. Conversely, insufficient vitamin D status was associated with higher odds of increased appetite compared to sufficient status in women with the CT genotype (OR, 1.78; 95% CI, 1.03, 3.07), and there was no association in women with the CC genotype (OR, 1.62; 95% CI, 0.89, 2.94). Premenstrual fatigue showed similar genotype-specific associations. Among women with the TT genotype, those with insufficient status had lower odds of experiencing premenstrual fatigue (OR, 0.44; 95% CI, 0.20, 0.97) than women with sufficient vitamin D status. However, in women with the CC genotype, insufficient vitamin D status was associated with significantly higher odds of experiencing premenstrual fatigue (OR, 2.53; 95% CI, 1.40, 4.56) compared to sufficient vitamin D status. In women with the CT genotype, vitamin D insufficiency and premenstrual fatigue were not associated. Although a significant interaction effect (*p* = 0.039) was found between *VDR* and vitamin D status on premenstrual nausea, no significant associations were observed when the analyses were stratified by genotype.

**Table 2:** Associations between inadequate vitamin D status and risk of premenstrual symptoms stratified by *VDR* genotype

`	Unadjusted OR	Unadjusted	Adjusted OR	Adjusted	Unadjusted OR	Unadjusted	Adjusted OR	Adjusted	Unadjusted OR	Unadjusted	Adjusted OR	Adjusted
	(95% CI)	*p*	(95% CI) ^a^	*p*	(95% CI)	*p*	(95% CI) ^a^	*p*	(95% CI)	*p*	(95% CI) ^a^	*p*
	CC				CT				TT			
Acne/skin blemish	0.89 (0.54, 1.48)	0.66	0.97 (0.54, 1.73)	0.91	0.83 (0.53, 1.32)	0.43	0.88 (0.52, 1.50)	0.63	0.67 (0.35, 1.29)	0.23	0.74 (0.35, 1.57)	0.44
Bloating/swelling/breast tenderness	1.18 (0.68, 2.07)	0.55	1.34 (0.71, 2.51)	0.37	0.60 (0.36, 1.00)	0.048	0.71 (0.39, 1.27)	0.24	0.77 (0.38, 1.58)	0.48	0.76 (0.34, 1.72)	0.51
Cramps	1.29 (0.74, 2.25)	0.38	1.17 (0.61, 2.24)	0.63	0.81 (0.48, 1.36)	0.42	0.93 (0.50, 1.74)	0.82	1.26 (0.53, 3.01)	0.60	1.88 (0.66, 5.33)	0.24
Mood swings/crying easily/irritability/angry outbursts	1.03 (0.57, 1.85)	0.92	1.08 (0.55, 2.11)	0.83	0.85 (0.52, 1.41)	0.54	0.88 (0.50, 1.58)	0.68	0.93 (0.47, 1.84)	0.84	0.89 (0.41, 1.94)	0.77
Increased appetite/food cravings	1.37 (0.81, 2.30)	0.24	1.62 (0.89, 2.94)	0.12	1.56 (0.98, 2.47)	0.060	1.78 (1.03, 3.07)	0.040	0.53 (0.27, 1.05)	0.070	0.48 (0.22, 1.04)	0.06
Fatigue	1.91 (1.15, 3.17)	0.013	2.53 (1.40, 4.56)	0.0021	1.06 (0.68, 1.67)	0.79	1.12 (0.66, 1.90)	0.67	0.63 (0.33, 1.20)	0.16	0.44 (0.20, 0.97)	0.04
Headache	0.87 (0.51, 1.48)	0.61	0.99 (0.53, 1.84)	0.98	1.36 (0.78, 2.37)	0.28	1.48 (0.78, 2.82)	0.23	0.63 (0.30, 1.30)	0.21	0.39 (0.15, 1.00)	0.05
Anxiety/tension/nervousness	1.16 (0.70, 1.91)	0.57	1.63 (0.92, 2.91)	0.097	1.38 (0.87, 2.21)	0.18	1.39 (0.81, 2.38)	0.24	0.80 (0.41, 1.60)	0.54	0.63 (0.28, 1.42)	0.27
Clumsiness	1.08 (0.58, 2.00)	0.81	1.55 (0.77, 3.15)	0.22	1.35 (0.68, 2.68)	0.39	1.49 (0.68, 3.29)	0.32	1.11 (0.44, 2.82)	0.82	0.88 (0.90, 2.62)	0.82
Confusion/difficulty concentrating/forgetfulness	1.58 (0.89, 2.79)	0.12	2.11 (1.09, 4.06)	0.026	2.10 (1.18, 3.76)	0.012	2.13 (1.10, 4.13)	0.026	2.22 (0.95, 5.24)	0.067	2.07 (0.78, 5.48)	0.14
Sexual desire/activity changes	1.34 (0.82, 2.21)	0.25	1.99 (1.09, 3.63)	0.025	1.04 (0.66, 1.65)	0.86	1.26 (0.73, 2.18)	0.41	1.10 (0.59, 2.08)	0.76	1.24 (0.59, 2.62)	0.57
Insomnia	1.46 (0.68, 3.16)	0.33	2.23 (0.92, 5.41)	0.077	0.73 (0.35, 1.51)	0.39	0.61 (0.26, 1.41)	0.24	1.63 (0.61, 4.33)	0.33	1.15 (0.38, 3.55)	0.80
Nausea	1.87 (0.88, 3.95)	0.10	2.44 (1.00, 5.95)	0.050	0.53 (0.27, 1.04)	0.067	0.59 (0.27, 1.28)	0.18	1.06 (0.45, 2.50)	0.90	1.48 (0.52, 4.16)	0.46
Depression	1.31 (0.75, 2.28)	0.34	1.84 (0.97, 3.48)	0.063	1.37 (0.83, 2.25)	0.22	1.63 (0.90, 2.94)	0.11	0.71 (0.36, 1.43)	0.34	0.67 (0.30, 1.49)	0.32
Desire to be alone	1.51 (0.89, 2.56)	0.13	2.22 (1.19, 4.13)	0.012	1.51 (0.94, 2.45)	0.092	1.43 (0.82, 2.48)	0.21	1.12 (0.58, 2.13)	0.74	1.14 (0.54, 2.42)	0.74

^a^Models were adjusted for age, ethnicity, BMI, physical activity, season of blood draw, calcium intake, and use of analgesics

**Table 3:** Interaction between *VDR* and vitamin D status on premenstrual symptoms

Premenstrual symptom	***VDR*** and vitamin D status interaction ***p***^**a**^
Acne/skin blemish	0.78
Bloating/swelling/breast tenderness	0.19
Cramps	0.39
Mood swings/crying easily/irritability/angry outbursts	0.88
Increased appetite/food cravings	0.027
Fatigue	0.016
Headache	0.25
Anxiety/tension/nervousness	0.44
Clumsiness	0.91
Confusion/difficulty concentrating/forgetfulness	0.82
Sexual desire/activity changes	0.60
Insomnia	0.22
Nausea	0.039
Depression	0.23
Desire to be alone	0.70

^a^*P* values for the interaction between *VDR* and vitamin D status were adjusted for age, ethnicity, BMI, physical activity, season of blood draw, calcium intake, and use of analgesics

## Discussion

The present study investigated the modifying effect of *VDR* genotype (rs7968585) on the association between insufficient vitamin D status and the presence of 15 symptoms commonly experienced by women prior to menstruation. Our findings are to be interpreted with caution because none of the vitamin D-genotype interaction effects on specific symptoms met our threshold for multiple comparisons. Nevertheless, the present study’s results suggest that a common *VDR* polymorphism may modify the association between vitamin D status and premenstrual increased appetite, fatigue, and nausea. Participants homozygous for the T allele and with insufficient vitamin D status had decreased odds of experiencing premenstrual fatigue and increased appetite, while subjects homozygous for the C allele and with insufficient vitamin D had increased odds of experiencing premenstrual fatigue and nausea compared to those who were vitamin D sufficient, while women who with the CT genotype and with insufficient vitamin D status had increased odds of experiencing premenstrual increased appetite. To our knowledge, this is the first study to examine the interaction between variation in the *VDR* gene, vitamin D status, and individual PMSx.

Inverse associations between vitamin D status or plasma 25(OH)D concentrations and PMSx have been reported previously [14, 15]. Our earlier analysis of the TNH population showed that women with inadequate vitamin D status had increased odds of experiencing several common premenstrual symptoms, compared to vitamin D-sufficient women [15]. Similarly, a case-control study nested in the Nurses’ Health Study II found an inverse association between 25(OH)D concentrations and premenstrual depression, diarrhea, fatigue, and breast ache [14]. Other studies, however, found no associations between plasma 25(OH)D and PMS or individual symptoms [32–34]. Although several reasons may explain these discrepancies, as described previously [15], the potential influence of genetic variation has remained unexplored. Therefore, the present study aimed to expand on our previous findings on the effects of vitamin D on PMSx in the TNH population by examining whether genetic variation in the *VDR* gene modified any associations.

In the present analysis, women who had insufficient vitamin D status and were homozygous for the C allele of the rs7968585 variant had significantly increased odds of experiencing premenstrual fatigue and nausea. It was previously found that the rs7968585 variant modified the association between low vitamin D concentration and risk of major health outcomes where the replacement of the T allele with the C allele conferred stepwise increases in risk in those with low 25(OH)D [22]. The association between low vitamin D status and PMSx is in keeping with predicted physiological mechanisms, as described in our previous publication [15], and perhaps homozygosity of the minor allele of rs7968585 may magnify these effects. However, our finding that T allele homozygotes with insufficient vitamin D status had reduced odds of experiencing certain PMSx compared to those who were vitamin D sufficient was unexpected.

The directionality of the associations between vitamin D and PMSx in CC homozygous participants is that which would be expected, based on observed physiological mechanisms that take place in premenstrual disorders. Vitamin D’s ability to normalize calcium fluctuations and regulate calcium homeostasis throughout the menstrual cycle has been hypothesized to protect women against premenstrual symptoms [35]. Furthermore, the role of vitamin D in reducing inflammation may provide a potential mechanism for its mitigation of specific PMSx. Elevated inflammatory markers have been previously observed in women with PMS [36], and vitamin D’s anti-inflammatory effects may help ameliorate the inflammation leading to PMSx.

To exert its physiological effects, the vitamin D-VDR complex forms a heterodimer with retinoid X receptor within the cell nucleus and binds vitamin D responsive elements as a transcription factor for multiple target genes. Single nucleotide polymorphisms (SNPs) present in the *VDR* gene alter receptor length, thus affecting its activation of target cells [37]. The physiological relevance of the nucleotide substitution in rs7968585 is not known; however, as in the case with other SNPs within *VDR*, it is possible that the T allele of rs7968585 upregulates downstream pathways that have adverse effects on health upon binding to a sufficient amount of vitamin D. For example, a G>C variant in a common *VDR* polymorphism, FokI, results in a VDR protein that is three amino acids shorter. This shorter isoform has been shown in transfection experiments to produce a more potent immune response with higher nuclear factor kappa-B and interleukin 12 expression [38]. Although further research is needed to elucidate the effects of the rs7968585 variant, it is possible that the TT genotype could confer increased risk toward PMSx in response to activation by vitamin D through activation or repression of genes that increase or decrease the risk of certain PMSx, respectively. This could help explain our observation that T allele homozygotes with insufficient vitamin D status had reduced odds of experiencing certain PMSx compared to those who were vitamin D sufficient. These findings further highlight the inter-individual variation in PMSx, suggesting that vitamin D may either have beneficial or unfavorable effects on PMSx based on variation in the *VDR* gene. This may help health care providers and researchers identify the discrepancy in the effectiveness of vitamin D in PMSx management.

The present study had several strengths. Participants were part of Canada’s three major ethnic groups, and findings could thus be generalized to a broader population of young Canadian adults. Moreover, the large sample size allowed us to investigate the relationship between vitamin D status and individual PMSx, instead of the somewhat arbitrarily defined disorders, PMS and PMDD. The GHLQ also collected detailed information concerning a subject’s lifestyle choices and current state of health, which allowed for adjustment for factors known to influence PMSx and vitamin D status, and thus, minimized the effect of residual confounders. Stratification by genotype further reduces risk of confounding influence on study findings.

There were also limitations which may affect the interpretation of the present results. This study was a cross-sectional analysis, and any observed associations cannot imply causation. Additionally, a retrospective questionnaire was used to report PMSx severities, which may result in overestimation of the prevalence of some symptoms. However, recall bias is unlikely to have differed by vitamin D status or by genotype. Additionally, we were unable to assess the effect of vitamin D binding protein concentrations and other vitamin D metabolites on the outcomes of interest. Subjects were also young adults recruited from a large university campus, and results may not be representative of all menstruating women. A greater understanding of the physiological effects of rs7968585 would facilitate clinical interpretation of this study’s findings. We also did not examine other variants in *VDR* or variants in other genes that affect the metabolism of vitamin D, such as *CYP27B1*, which is involved in hydroxylation of 25(OH)D to 1,25(OH)D [39] and may influence the relationship between vitamin D and PMSx.

In summary, the findings of the present study suggest that vitamin D may have either beneficial or detrimental effects on PMSx based on variation in the *VDR* gene. However, these results must be interpreted with caution because the identified *VDR-*vitamin D interactions did not meet our threshold for multiple comparisons. More studies with a larger sample size that investigate not only rs7968585 within *VDR* but also variants within other key vitamin D metabolism genes, are needed to help elucidate the relationship between vitamin D and PMSx.

## Supplementary Information

**Additional file 1: Supplementary Table 1.** Ethnic groups stratified by *VDR* rs796858 genotype^a^

## Data Availability

The datasets used and analyzed during the current study are available from the corresponding author upon reasonable request.

## Abbreviations

PMSx: Premenstrual symptoms
VDR: Vitamin D receptor
(25(OH)D): Plasma 25-hydroxyvitamin D
PMDs: Premenstrual disorders
PMS: Premenstrual syndrome
PMDD: Premenstrual dysphoric disorder
(1,25(OH)D): 1,25-dihydroxyvitamin D
TNH: Toronto nutrigenomics and health
GHLQ: General health and lifestyle questionnaire
FFQ: Food frequency questionnaire
HC: Hormonal contraceptives
BMI: Body mass index
MET: Metabolic equivalent
OR: Odds ratios
CI: Confidence interval
SNPs: Single nucleotide polymorphisms
