# Correction to: Variation in the vitamin D receptor gene, plasma 25-hydroxyvitamin D, and risk of premenstrual symptoms

**DOI:** 10.1186/s12263-021-00699-z

**Published:** 2021-10-19

**Authors:** Alicia C. Jarosz, Daniel Noori, Tara Zeitoun, Bibiana Garcia-Bailo, Ahmed El-Sohemy

**Affiliations:** Department of Nutritional Sciences, Temerty Faculty of Medicine, University of Toronto, Toronto, Ontario M5S 1A8 Canada

**Correction to: Genes Nutr. 16, 15 (2021)**

**https://doi.org/10.1186/s12263-021-00696-2**

Following publication of the original article [1], it was brought to our attention that the article had published with incomplete equal contributions information: the third author, who contributed equally along with the first and second authors, had not been marked as contributing equally.

The original article has since been updated and the corrected author list can be found in this correction.

The publisher apologizes for this processing error.
