# Supplementary material for: Variation in the vitamin D receptor gene, plasma 25-hydroxyvitamin D, and risk of premenstrual symptoms
Source: Genes Nutr. 2021 Sep 22;16:15. doi: 10.1186/s12263-021-00696-2 (PMC8459465; doi:10.1186/s12263-021-00696-2)
Supplement: Supplementary file 1 — Additional file 1: Supplementary Table 1. Ethnic groups stratified by VDR rs796858 genotypea [file 12263_2021_696_MOESM1_ESM.docx]

**Supplementary Table 1.** Ethnic groups stratified by *VDR* rs796858 genotype ^a^

|  | **CC** | **CT** | **TT** | ***p* value** ^a^ |
| --- | --- | --- | --- | --- |
| Total Population, n (%) | 258 (36) | 303 (42) | 155 (22) |  |
| Ethnicity |  |  |  | <0.001 |
| Caucasian | 51 (20) | 124 (49) | 79 (31) |  |
| East Asian | 177 (54) | 122 (37) | 31 (9) |  |
| South Asian | 16 (20) | 38 (46) | 28 (34) |  |
| Other | 14 (28) | 19 (38) | 17 (34) |  |

^a^ Differences between groups were compared using chi-square tests
